# Conversion of a potentiometric analyser to control by the ZX Spectrum microcomputer

**DOI:** 10.1155/S1463924685000463

**Published:** 1985

**Authors:** S. W. Bateson, G. J. Moody, J. D. R. Thomas

**Affiliations:** Department of Applied Chemistry Redwood Building UWIST Wales Cardiff CF1 3XF UK


Journal of Automatic Chemistry/*Journal of Clinical Laboratory Automation*, Vol. 7, No. 4 (October–December 1985), pp. 206–207

### Conversion of a potentiometric analyser to control by the ZX Spectrum microcomputer

**S. W. Bateson, G. J. Moody and J. D. R. Thomas**
*Department of Applied Chemistry, Redwood Building, UWIST, Cardiff CF1 3XF, Wales, UK*

### Introduction

A potentiometric titration-calibration system based around the Sinclair ZX 81 microcomputer has previously been described [1]. Although this proved adequate as a system controller, it had two drawbacks:

(1) Experimental data could not be stored on tape without recording the whole program, which took over 10 min, and data storage of each experiment was, therefore, not practical.
(2) Graph plotting on the X-Y plotter was slow (3 to 4 min per plot), while the low screen resolution prevented useful display on the screen.

The above problems have been overcome by use of the Sinclair ZX Spectrum which, besides such benefits as much faster processing and more memory, allows for graph plotting on screen with relatively high resolution (256 × 176 points) and the saving of titration data, independently of the main program, in 15–20 s. Two modifications have been made to the earlier [1] interface circuitry in order to adapt it to the Spectrum.

### Interface system modifications

The alterations involved replacing the address decoder and simplifying the digital switch decoder.

As previously mentioned [1], the Z80 CPU can address both memory and input/output locations. As the Spectrum has 'IN' and 'OUT' commands on its keyboard (these being the I/O equivalent of PEEK and POKE), it was found convenient to 'I/O map' the interface. This considerably simplifies the address decoder (figure 1).

In I/O operations, the Spectrum uses the lowest four address lines but not the next four (unless Microdrives are connected) so the address decoder simply detects an I/O request for an address other than those involving the bottom four address lines (figure 1). Since no memory space is taken up, no read/write switch (figure 3 in [1]) is needed. The data-bus buffer remains, however.

The digital switch decoder (figure 2) was simplified to perform the four essential functions; these are given in table 1.

The decoder in figure 2 is a two-to-four line decoder; the data latch and level-shifting circuits are otherwise unchanged.

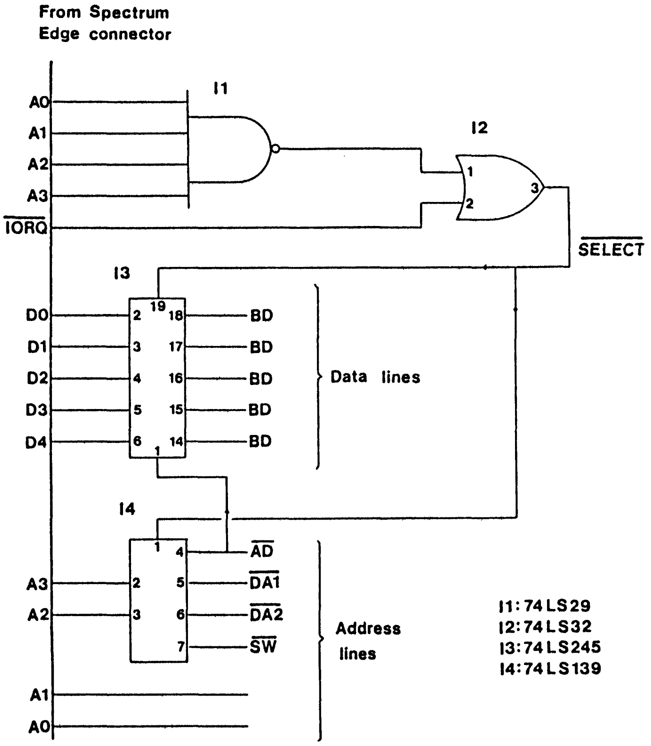

*Figure 1. Spectrum address decoder.*

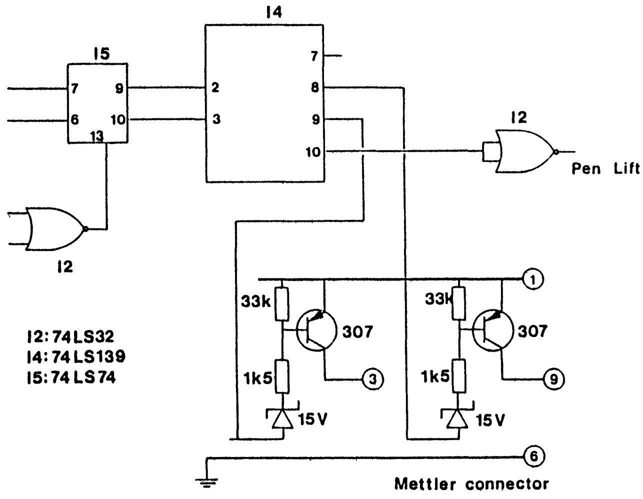

*Figure 2. Digital switch decoder for the Spectrum.*

*Table 1. Revised digital switch function.*

| Function | *N* | IC4B pin |
|---|---|---|
| Normal (no function) | 0 | 7 |
| Add 1 mm$^3$ from burette | 1 | 8 |
| Add 0.1 cm$^3$ from burette | 2 | 9 |
| Lower chart-recorder pen | 3 | 10 |

### Program

The titration program is more comprehensive than the ZX 81 program, but the basic structure and features remain the same. However, a few points are mentioned here:

(1) The titration routine continuously displays the progress of the experiment on-screen. Titrant addition volume is decided by predicting the titration end-point and adding a fixed fraction of the estimated remaining titrant volume. This approach was found more efficient than the earlier one [1], which was disrupted by the extreme e.m.f. changes found at sulphide and thiol titration end-points for which the titration was used [2]. The end-point volume is estimated by a subroutine which includes the equation:

$$V_{EP} = V_1 + \frac{V_1 - V_0}{1 - 10^{(E_1 - E_0)/S}}$$

where $V_{EP}$ is the estimated endpoint volume, $V_1$ and $V_0$ are the current and preceding titrant volumes, $E_1$ and $E_0$ the current and preceding cell e.m.f.s and $S$ is the slope of the electrode response. The estimated end-point is displayed after each addition.

(2) When the titration is completed, all graphs can be displayed on the screen. The *x*-axis can be expanded to 'zoom-in' on details, and any resulting display copied to the X-Y plotter.

(3) An indicator electrode calibration curve can be generated before or after the end-point, to show response to titrand or titrant. Also, the slope and intercept of (selected portions of) the calibration curve can be calibrated and displayed.

### Conclusion

Adaptation of the previously described microcomputerized potentiometric analyser [1] to the Sinclair ZX Spectrum microcomputer has greatly increased the versatility of the system. This has been successfully used for titrating suphur containing components with silver nitrate and mercury(II) chloride titrants in oil refinery process stream samples [2].

### Acknowledgements

The authors thank the Science and Engineering Research Council for a studentship (to SWB) under the CASE scheme in conjunction with the Esso Petroleum Company PLC, Abingdon, UK, and especially to Mr D. Murtagh for his encouragement.

### References

1. Bateson, S. W., Moody, G. J. and Thomas, J. D. R., *Journal of Automatic Chemistry*, **5** (1983), 174.
2. Bateson, S. W., Moody, G. J. and Thomas, J. D. R., *Analyst*, in the press.

**SAC 86/3rd BNASS: AN INTERNATIONAL CONFERENCE ON ANALYTICAL CHEMISTRY AND ATOMIC SPECTROSCOPY**

To be held from 20 to 26 July 1986 at the University of Bristol, UK, SAC 86/3rd BNASS is organized by the Analytical Division of the Royal Society of Chemistry in conjunction with the Spectroscopy Group of the Institute of Physics.

Plenary lectures, which will be published in *The Analyst,* include:

Professor J. H. Knox (University of Edinburgh, UK):
'Advances in columns and packing for high performance liquid chromatography'.
Professor M. Bonner Denton (University of Arizona, Tucson, USA):
'Concepts for improved automated laboratory productivity'.
Professor B. V. L'Vov (Polytechnic Institute, Leningrad, USSR):
'New advances in furnace atomic absorption spectrometry'.
Professor G. Tölg (Max Planck Institute for Metal Research and Institute for Spectrochemistry, Dortmund, FR Germany):
'Extreme trace analysis of the elements – the state of the art today and tomorrow'.

The scientific work of the conference will be centred round the presentation and discussion of contributed papers on all aspects of analytical chemistry and applied spectroscopy. The programme will consist of accurately-timed parallel lecture sessions and all-day poster presentations arranged in various themes based on techniques, materials and other aspects of analysis.

*More information from Miss P. E. Hutchinson, Secretary of the Analytical Division, Royal Society of Chemistry, Burlington House, London W1V 0BN.*

